# A QALY is [still] a QALY is [still] a QALY? Evaluating proportional shortfall as the answer to the problem of equity in healthcare allocations

**DOI:** 10.1186/s12910-024-01036-w

**Published:** 2024-03-23

**Authors:** Hamideh Mahdiani, Nikolai Münch, Norbert W. Paul

**Affiliations:** Institute for History, Philosophy and Ethics of Medicine, Johannes Gutenberg University Medical Center, Am Pulverturm 13, 55131 Mainz, Germany

**Keywords:** Cost-effectiveness, Biomedical Ethics, Equity, cancer

## Abstract

Despite clinical evidence of drug superiority, therapeutic modalities, like combination immunotherapy, are mostly considered cost-ineffective due to their high costs per life year(s) gained. This paper, taking an ethical stand, reevaluates the standard cost-effectiveness analysis with that of the more recent justice-enhanced methods and concludes by pointing out the shortcomings of the current methodologies.

## Introduction

Governments and Health policy makers in many Western countries rely on the recommendations of institutional, often national, advisory boards to establish health priority setting and resource allocation, one source of which is the evidence provided by cost-effectiveness analysis (CEA). As a popular economic evaluation technique, CEA compares the costs and benefits of interventions—the value for money—that are intended to enhance health [1] and to optimize outcomes. The standard approach frequently employed in CEA involves assessing quality-adjusted life years (QALYs) in conjunction with the incremental cost-effectiveness ratio (ICER). This approach evaluates the impact of introducing a new intervention on costs and QALYs, in comparison to existing options. It also considers society’s willingness to pay (WTP), which represents the maximum cost a particular society deems appropriate to invest in a single QALY. QALY scores (utility: 0 to 1, 1 being full health [2]) serve as a common currency even though they are applied in various circumstances. However, there is enough evidence that some QALYs are preferred over others and not all QALYs are valued equally by public perception^1^ [3−7], which often points at the lack of equity-based notions of justice under distributional effects of health interventions. Although it has been more than four decades that the role of equity in allocation decisions in the health care sector has been pointed out [8], and only few countries enforce strict QALY thresholds for reimbursement decisions irrespective of other considerations, the systematic inclusion of equity considerations in QALY scores are only slowly increasing [9]. In other words, it seems that, a QALY is [still] a QALY is [still] a QALY in economic evaluations [10] and health gains are valued equally [6, 11] within this metric.

Against this background and given the growing pressure on health care budgets, in this commentary we take an ethical stand towards this issue and provide an exemplary case in the treatment of non-small-cell Lung cancer (NSCLC) with an immunotherapeutic drug, namely Pembrolizumab (Pem), as monotherapy or in combination with other therapies. We explore and explain why the standard CEA needs to be modified in the context of improving patient outcome and how justified current approaches still can be. To answer this, we are presenting the case using available reports from standard CEA scores (the norm in most parts of the world) and equity-weighted CEA (the standard in countries like Norway, the Netherlands or UK). We choose to focus on lung cancer as it is the number one cancer-related mortality cause worldwide and has a five-year survival rate of less than 20%. To improve patient outcome, recent therapeutic modalities like (combination) immunotherapy are being developed and offer hope for new and more effective treatments. Scientists posit that due to the diverse nature of NSCLC, treatment should be tailored to each patient’s unique clinical condition, including factors such as performance status, disease stage, histological cell type, and molecular profile [12]. In the context of economic evaluation, it is worth noting that cancer therapies, in general, tend to be considerably less cost-effective compared to treatments for non-cancer conditions [13]. Healthcare policymakers often perceive these treatments as too expensive for the healthcare system, resulting in limited access for patients – the question is if this is ethically appropriate. Our commentary concludes by highlighting the ethical challenges, which the current equity-weighing CEAs can, and do pose and by maintaining that a better patient outcome demands not only medical attention but also a suitable CEA.

## CEAs: Standard and enhanced

Traditional CEA still focuses on “economic efficiency by prioritizing health care interventions that maximize health gains across a population within a given budget” [14]. For example, study reports from the United States are mostly in favor of Pem in combination with chemotherapy for patients with PD-L1 level more or equal to 50% (PD-L1 >/=50%) [15–16], which is contrary to results from China [17] where results showed that, compared with chemotherapy, the combination strategy is cost-ineffective for the treatment of NSCLC in the American and Chinese health care system at WTP threshold of $100,000 per QALY for the United States and $27,351 per QALY for China. In the same manner, using a WTP threshold of 50,000 £, researchers [17] show that Pem is not cost-effective in the UK at its current list price and a discount of 50% or more is required for it to be cost-effective compared to commonly prescribed chemotherapy. Others [18], also from the American context, present similar results with a focus on the cost-effectiveness of first-line Pem for patients with various comorbidities where Pem is still cost-ineffective compared to chemotherapy. Pem was reported cost-effective for first-line treatment of PD-L1-positive (50%) metastatic NSCLC patients in France, with a WTP threshold of 100,000€/QALY [19].

These CEAs, however, only evaluate the overall efficiency of an intervention in maximizing health (in this context that is QALYs) regardless of how those benefits in health are distributed across the population. However, in many cases, considering only efficiency (and implicitly assuming “distributive neutrality” [5]) violates some widely held intuitions about how to justly allocate health care resources. For example, a relatively inexpensive therapy for a widespread but mild condition, which does not involve much QALY losses per person might be more cost effective than a cancer therapy using immunotherapy. Nevertheless, many people might intuitively feel that it might be more ethically appropriate to pay for the therapy for the cancer patients instead of paying for the mild widespread condition. This moral intuition hinges on a common sense that cancer patients are worse off than the patients with mild conditions and that equity in healthcare has something to do with giving at least some priority to those worse off. This idea – equity as (among other things) improving the position of the worse off – is also very common in medical ethics. Nevertheless, there are considerable differences in concretizing what it means to be worse off and in spelling out how the benefits for the worse off have greater weight (that is how the trade-off between efficiency and equity is arranged^2^).

## A closer look at equity and shortfall

In order to promote a more equitable and fair distribution of healthcare resources, equity weights can be applied to health gains or taken into account in the monetary threshold [6, 20]. As certain demographic groups may have disparities in health outcomes and access to care, equity-weighted cost-effectiveness analysis attempts to address the aforementioned inequities in the decision-making process.  It attempts to prioritize interventions that not only maximize health benefits but also promote justice and lessen health inequities by giving various weights to health gains based on equity considerations. For the sake of the argument, we focus on two such methods [21, 22], namely *proportional shortfall* (PS) and *absolute shortfall* (AS), utilized in practice in the Netherlands and Norway respectively (for a systematic review on justice concern in CEA cf. [42]). Both methods are meant to spell out which persons are worse off and should therefore be given some priority when it comes to the allocation of resources. For both considerations, the severity of disease is the central aspect of what it means to be worse off. Albeit PS and AS have different approaches in fleshing out what it means to have a severe disease, both methods already have an impact on the CEA of cancer therapies.

In PS, the differentiated QALY produces a score (i.e., a PS score [23]) between 0 (no health loss) and 1 (full health loss or death) based on the severity of the disease [5]. This method expresses the average health loss resulting from a condition over the patient’s remaining lifetime in QALYs as a proportion of the total potential health that the patient could have had without the condition [24]. It has been argued that PS balances societal concerns as it encompasses elements used to derive the absolute shortfall, fair innings, and rule of rescue [24] - and it equalizes relative benefits between persons with respect to their potential for health [25]. Literature [5, 7, 26] shows that using PS in the Netherlands has improved the shortcomings associated with the standard CEA in some ways by quantifying health losses in terms of QALYs, making it applicable to different diseases and patient populations. Schurer et al. [24] have additionally proven that the inclusion of PS in the CEA has changed the Dutch National Healthcare Institute’s (Zorginstituut Nederland [ZIN]) recommendations. For example [27], in the package advice for Pem for the treatment of NSCLC, with a PS of 0.7 to 0.9 and with price negotiations, Pem has been recommended for reimbursement.

This is different from the Norwegian approach where *absolute shortfall* (AS) is calculated as the disease-related loss of remaining QALYs without the new health technology, compared to the remaining QALY expectation in absence of the disease [6]. In Norway, extra priority is given to patients with larger loss of prospective health (i.e., low health-adjusted life expectancy), emphasizing a key dimension of the severity of a disease, defined as the mean absolute shortfall of QALYs in the patient group receiving standard care compared with a reference group of healthy persons of a similar age [28]. For interventions addressing more severe disease categories, the government accepts a higher threshold for willingness-to-pay per QALY. Tranvåg et al.’s [29] study on “Appraising Drugs Based on Cost-effectiveness and Severity of Disease in Norwegian Drug Coverage Decisions” shows that the severity-adjusted ICER best projected a positive drug coverage decision with Cancer drugs being the most frequently appraised drugs, representing 113 of 188 (60%) of decisions in the period between 2014 and 2019.

It seems that the same trend is spreading in other European countries. In the UK, new methods manual for NICE’s health technology assessments will replace the end-of-life criterion with a severity modifier, meaning that treatments with greater absolute or proportional QALY shortfalls should be prioritized [29, 30]. With this new approach, incremental QALY gains from new medicines will be given more weight if the illness severity is high enough [29, 31]. Are then PS or AS the answer to the equity-related concerns in the standard CEA?

## Equity and justice

Selecting an appropriate metric requires a well-justified decision that involves explicit value judgments, especially in light of several complex conceptual issues. It should be recognized that different equity metrics might yield divergent results and conclusions. First, we need to distinguish between two ways of understanding equity: horizontal and vertical. Horizontal equity means that people with the same health needs should have the same access to health services, regardless of other factors such as income, social status, or geographical location. Vertical equity, on the other hand, recognizes that people with different health needs should be treated differently to reduce health inequalities. For example, people with serious illnesses or chronic conditions may require more extensive and expensive health services.

PS typically supports the concept of vertical equity in healthcare, meaning that people with greater healthcare needs receive a proportionally greater share of resources or attention. Why does this matter? Ethically, it matters because vertical equity would involve directing more resources to those with serious or chronic illnesses to address the shortfall in their healthcare needs, which can cause some ethical issues.

A notable concern is that the reliance on PS could lead to a system that is biased in favor of terminal illnesses [5, 32]. PS also underscores the inherent discrimination against younger individuals, who may suffer a substantial loss of quality-adjusted life years (QALYs) only to find that this is offset by a much smaller loss of QALYs [32]. This is in line with Van de Wetering et al.‘s [33] argument against PS. They claim that PS has a rather counterintuitive implication, particularly in the context of imminent death. In particular, the principle assigns a need score of 1 regardless of the absolute number of years of life lost, showing indifference to whether a 3-year-old loses 80 years or an 80-year-old loses 3 years (assuming both are expected to live to 83). In practice, however, many people tend to see the first scenario as more important to intervene in, potentially contradicting societal principles of equity particularly when it comes to addressing health needs and preventive strategies in the broader population.

Similarly, Richardson et al. [32] suggest that equity should be assessed explicitly and through variables related to age and severity, rather than relying on PS alone. While PS and AS both apply adopted WTP thresholds according to the different categories of severity of disease, they can’t consider other individual factors that may have an impact on the objective and subjective outcomes of therapy in question (that is on the QALY of that individual patient). As mentioned above, cancer therapy is one leading field of precision medicine. Tailoring therapies to the special needs of individual patients (e.g. molecular profile, genetic dispositions or clinical condition) significantly improves outcomes (but may also rise costs [34, 35]). CEAs with or without PS or AS in contrast are not suited to reflect the level of individualization of many personalized therapies because the result of the CEA (with or without PS or AS) are based on a probabilistic notion of an average patient within a larger group. Nevertheless, there will be patients that, for whatever reason, have the prospect of a therapy outcome above average (because of their genetics, age, life style factors, etc.). In addition, there will be patients, which evaluate the outcome as more valuable to themselves (in terms of QALYs) than the average. Both factors could lead to an individual CE-ratio (with or without PS/AS) that is better than the general CE for that therapy. Those patients would profit more of a certain therapy at a given cost than the average patient who is presupposed in general CEA but may be denied reimbursement because the reimbursement decision is made based on the average patient. So, even if PS and AS incorporate some equity concerns, those methods cannot keep step with more and more personalized therapies. Therefore, there is a risk that the gap between individual and average QALY and CE-ratio outcome will increase in times of individualized medicine. If this gap gets bigger the ethical concerns grow that CEAs even with incorporated equity concerns (with PS or AS) unduly lead to withholding therapy for patients which could benefit from them at a reasonable cost.

The growing stratification of some therapeutical approaches – especially in cancer therapy [36] - could be reflected by including more subgroup analysis in CEAs, which reflect the heterogeneity of patients and their differentiated outcome prospects. However, recent research states that the “ability of current modeling approaches to capture patient and treatment effect heterogeneity is constrained by their limited flexibility and simplistic nature” [37]. While more differentiated CEA analysis could improve reimbursement decisions, the general problem - that decisions on population level won’t hardly ever be able to do justice to every individual patient- remains. Maybe the subject of justice is - in Rawls famous words - the basic structure of society and not individual choice or action [38]. Nonetheless, simplistic CEA decisions falling behind the growing complexity of therapeutic approaches are an open ethical problem beyond any utopian notion of justice.

## Real world impact

The problem of equity even aggravates if we consider not only the distribution of health care resources but of health opportunities. There is a growing insight in the determinants of health where access to health care resources is but one variable of many. Even in affluent regions where there is ostensibly equitable healthcare access, disparities persist due to factors such as transportation expenses, cultural influences, and socio-economic status. Consequently, addressing healthcare equity necessitates a comprehensive approach that considers both geographical and income-related determinants.

There is also the issue of transparency. Novel drug discovery is anticipated to increase health care expenses in the years to come [29]. The price of new pharmaceuticals may be negotiated while considering their cost-effectiveness and how their health benefits are divided [29]. However, negotiated drug prices are typically confidential. As a result, it is difficult for the public to judge whether the criteria are followed in practice and to what extent access to new treatments is equitable and fair because information about list prices for drugs does not reflect actual prices, which highlights a lack of procedural fairness, as has been the case in Norway [29]. Furthermore, in a recent study of systemic anti-cancer therapy patterns in advanced non-small cell lung cancer in Europe, [39] it has been reported that resources for diagnostic testing, long reimbursement timelines and slow adoption of new medicines in clinical practice are still the main problems in the fight against NSCLC. Overall, an underuse of both immunotherapy and targeted therapy in nearly all European countries has been reported due to delay in reimbursements (6 months to 2 years) [39].

## Conclusion

As mentioned above, in the UK, the Netherlands and Norway, more equity-conscious strategies are being used with the consequence of adopting alternative (i.e., higher) cost-effectiveness thresholds [4, 5, 40] for some diseases and patient groups. In doing so, these nations acknowledge that distributive concerns —the value of a QALY as it relates to who receives benefits and when—needs to be incorporated in CEAs [41]. Nevertheless, the issue of relative value of health gains in relation to empirical estimates of marginal cost-effectiveness of current care remains indeed an understudied topic [26] and present equity-adjustments still face ethical questions. If CEAs- with equity adjustments- will ever be able to incorporate all or most important ethical concerns about justice in health care remains in doubt. Alternative accounts that incorporate equity concerns besides CEAs in turn run the risk of loosing the transparent and (arguably relatively) objective criteria reflected in CEA .

All being said, we believe that maximizing the level of health in society is unlikely to be driven by ethical deliberation alone. Health cannot be directly redistributed among members of society [43] due to economical, technological, and political restraints, which we need to address more openly even in countries of higher income with well-established health care systems. This implies at the same time, that this issue is even more demanding on the level of global health and justice particularly when willingness to pay is limited by the ability to pay.

## Data Availability

Not applicable.

## Footnotes

1 One key distinction to make is between formal health assessments, which aim for equitable QALY distribution, and policies that prioritize specific public health issues like cancer care or mental health. Flexibility in healthcare decisions is crucial, recognizing that a uniform approach to cost-effectiveness thresholds may not suit all conditions or contexts.

2 Prevention of severe diseases or early treatment of mild (and potentially progressing) diseases could be more cost-effective than treatment of actual severe diseases. While dedicating more resources to prevention could reduce the number of people suffering severe diseases like lung cancer, it would mean a (relative) reduction of resources available for treatment of this and other severe diseases. While such a shift to prevention could be cost effective and maybe even reduce inequity by preventing people getting worse off, it nevertheless would possibly go against the ethical intuition of helping people that are actually worse off (including but not limited to the rule of rescue). The balance of treatment and prevention (potentially including social measures that might reduce health inequity) is an important and debated topic but beyond the scope of our argument in this paper.
